# Characteristics Associated With Recruitment and Re-contact in Mayo Clinic Biobank

**DOI:** 10.3389/fpubh.2020.00009

**Published:** 2020-02-04

**Authors:** Matthew A. Hathcock, Christine Kirt, Euijung Ryu, Josh Bublitz, Ruchi Gupta, Liwei Wang, Stephen N. Thibodeau, Nicole L. Larson, Mine S. Cicek, James R. Cerhan, Janet E. Olson

**Affiliations:** ^1^Division of Biomedical Statistics and Informatics, Department of Health Sciences Research, Mayo Clinic, Rochester, MN, United States; ^2^Division of Epidemiology, Department of Health Sciences Research, Mayo Clinic, Rochester, MN, United States; ^3^Division of Digital Health Sciences, Department of Health Sciences Research, Mayo Clinic, Rochester, MN, United States; ^4^Department of Laboratory Medicine and Pathology, Mayo Clinic, Rochester, MN, United States

**Keywords:** biobanks, consent, participation, recruitment, follow-up study

## Abstract

**Objective:** To better understand the characteristics associated with a participant's willingness to consent to the Mayo Clinic Biobank (MCB) and examine factors associated with willingness to participate in follow-up studies embedded within MCB that require re-contact and participant approval.

**Participants and Methods:** Consent rates were compared across patient demographics to the MCB. Rates of participation to follow-up studies were also compared across demographics and request types.

**Results:** Among 272,102 Mayo Clinic patients invited to the MCB, 48,314 (19%) consented across the three recruitment sites within 90 days of initial invitation. A significant age by gender interaction was identified, showing young males consent at a lower rate than young females and older males consent at a higher rate than older females. Over the recruitment time frame of 2009–2015, there was a significant decrease in consent rates (decline of 2.5%/year). Of the 57,041 consented MCB participants, 33,487 participants (59%) have been invited to participate in follow-up studies via re-contact. Follow-up studies of the MCB may require participants to provide additional samples, complete questionnaires, and/or release their identity to a research team. MCB participants have been invited to enroll in a median of two studies (IQR: 1–3). Seventy-one percent of participants consented to at least one follow-up study, with individual follow-up study consent rates ranging from 14 to 87% depending on study type, with a median consent rate of 61% (IQR: 47–70%). Studies requesting return of a questionnaire had the highest participation rates. White participants, older participants, and participants with some college or a degree were significantly more likely to participate to follow-up studies, while there was no association with gender.

**Conclusion:** Consent rates among younger and non-white patients were lower than in older, white patients. However, we also found that participation rates among those already enrolled in the biobank were much higher than those seen in new recruitment efforts, external to an existing biobank. We thus demonstrate an important way that biobanks can advance precision medicine goals: through provision of populations from which studies can draw participants for future studies.

## Background

Biobanks are a key element in the advancement of precision medicine by facilitating research. The majority of biobanks accomplish this through the use of stored samples, but some also enable efficient re-contact and enrollment of subjects into new research projects. The Mayo Clinic Biobank (1) is one such collection that allows additional contacts for embedded follow-up studies. Understanding the factors related to participation in existing biobanks can aid in identifying patterns that predict recruitment to future collections. For example, the NIH funded *All of Us* study cohort is currently enrolling and has plans to allow future studies in the cohort (2). However, current biobanking literature has limited data on the rate at which subjects are willing to participate in additional embedded follow-up studies within biobanks to which they had consented. Understanding participant engagement in follow-up studies can further demonstrate the benefit of biobanking and increase efficiency in these types of recruitment. This study sought to investigate the factors associated with consent to the Mayo Clinic Biobank (MCB) as well as willingness to participate in additional follow-up research projects embedded in the MCB.

## Particpants and Methods

The MCB initiated recruitment of adult patients in April, 2009. Briefly, the initial recruitment phase identified patients through upcoming clinical appointments in Rochester, MN (MCR) starting in April, 2009. Minimal eligibility criteria were required for the MCB: Mayo Clinic Patients who were 18 or older, current residents of the United States, and able to provide informed consent. Appointments were selected largely from departments that provided primary care (1). The initial recruitment phase identified Mayo Clinic patients through the clinic appointment database in Rochester, MN (MCR) starting in April, 2009. Recruitment was expanded to the other Mayo Clinic sites in Jacksonville, FL (MCF) in June, 2012 and La Crosse, WI (MCW) in August, 2013. December, 2015 marked the end of active recruitment as the recruitment goal of enrolling 50,000 had been met. Since then, a passive enrollment continues as subjects approach the Biobank to request enrollment. One of the key elements included in the Mayo Clinic Biobank consent was allowance for up to two requests per year for additional studies (3). Over 40 follow-up studies with new patient contact have been conducted to date; each reviewed and approved by Mayo Clinic Institutional Review Board (IRB).

### Mayo Clinic Biobank Enrollment

In the original Biobank enrollment, we identified 272,013 Mayo Clinic patients with scheduled appointments up to 3 weeks in the future and mailed invitation packets to patient home addresses. Those who had not responded to the invitation were contacted via phone, unless the date of the scheduled appointment had passed. All subjects were given a remuneration valued at $20 after all required elements (consent, sample, survey) were completed. The 272,102 patients that were invited to the Mayo Clinic Biobank ranged in age from 18 to 95, with a mean age of 55 (SD: 17.4). Fifty-eight percent were female and 84% were of the white race. For the current analyses, we excluded 20,911 patients: 3,632 were identified to be ineligible (i.e., unable to provide informed consent), 6,853 were unsolicited volunteers, and 10,426 were subjects for whom we were unable to contact due to patient refusal of Minnesota Research authorization (Sec. 144.295 MN Statutes) (1). Among the remaining 251,102 patients, 48,312 participated, 23,944 refused, and 178,846 did not respond within 90 days. To understand consent rates, we compared subjects who participated to those that refused and did not respond. Responses to recruitment were truncated at 90 days to evaluate the initial recruitment and limit potential cofounding, such as additional recruitment contacts that occurred more than a year after the initial invitation. We evaluated differences in consent rates by basic demographic information: age at invitation, gender, race, ethnicity, distance from the clinic, and month of invitation. Other potentially important factors (such as education level) were unavailable to us due to IRB restrictions on accessing non-responder and refuser data.

### Follow-Up Studies Within Mayo Clinic Biobank

As described above, the MCB informed consent allowed contact of study participants up to two times per calendar year to participate in follow-up studies. These studies requested participants to provide additional samples (saliva, stool, blood, and/or urine), complete additional questionnaires, and/or release their contact information to the follow-up study to allow recruitment to a separate IRB approved protocol. Investigators conducting these follow-up studies provided selection criteria to the MCB staff who then subset to eligible participants through queries of patient provided information and/or electronic health record (EHR) data. MCB staff mailed the appropriate information to these participants outlining the request and describing the remuneration for the given study. Typically, follow-up studies offered remuneration in various amounts. Remuneration ranged from $0 for studies with only questionnaires, $10 for new samples to upwards of $800 for studies with greater subject burden (i.e., numerous clinic visits, invasive procedures, study diaries). Remuneration was mentioned at the time of the study invitation and distributed after all study requirements were complete.

Invited subjects indicated willingness to participate by completing an opt-in form and returning their response to the MCB study team personnel via the U.S. Postal service. Those who did not provide any response to the initial opt-in form were sent reminder letters 4 weeks after the initial mailing. All questions or clarifications received via mail, telephone, email, or in person were tracked in the study database. We evaluated difference in participation rates by study type and by basic demographic information: age at invitation, gender, race, ethnicity, education, and time from MCB consent to follow-up study invitation.

## Statistical Methods

Consent rates into the MCB were summarized as total number of consented participants divided by the total number of eligible subjects invited to participate within each category of a variable. Continuous variables (age, residential distance from the recruitment site, and years since MCB consent to follow-up study invitation) were categorized as follows: age in 20 year windows; distance from MCB was split into local (<30 miles), care in catchment area (31–99 miles), regional (100–249 miles), and national (250 or more miles). Time from initial Biobank consent was grouped into time frames with similar consent rates: <1, 2–3, and more than 3 years. Consent to the MCB was summarized across the three sites (Rochester, MN; La Crosse, WI; and Jacksonville, FL) separately and as a whole.

Participation rates to follow-up studies were summarized as weighted means by sample size to account for the multiple studies to which participants were invited to participate. Weights were calculated for each category of a variable across follow-up studies. Weights were calculated as number invited to a given study within a level divided by the total number of patients invited in that variables level to all sub-studies. Age/gender interactions were investigated using logistic regression with a splined age with 3 degrees of freedom (4). *P*-values were calculated but not presented for the majority of analyses in this study due to the large sample size which causes statistical significance for even trivial differences. Statistical analysis was performed using SAS software version 9.4 (SAS Institute Inc., Cary, NC) and R 3.1.1 (R Foundation for Statistical Computing, Vienna, Austria).

## Results

### MCB Participation

**Figure 1:**
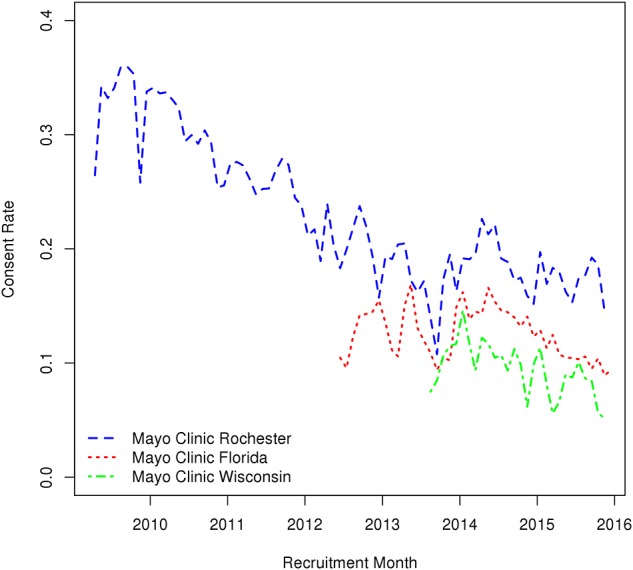
Variations in monthly consent rate to the Mayo Clinic Biobank during the Biobank enrollment period from April 1, 2009 through the end of 2015 at the three recruitment centers (MCR, Mayo Clinic Rochester; MCF, Mayo Clinic Florida; MCHS, Mayo Clinic Health System site in La Crosse, Wisconsin).

Of the 251,102 patients actively invited to participate in the Mayo Clinic Biobank between April 1, 2009 and the end of December, 2015, 48,312 (19%) consented, 23,944 (10%) refused, and 178,846 (71%) did not respond within 90 days of initial invitation. Mayo Clinic Rochester had the highest overall consent rate at 24% with Mayo Clinic Florida and Mayo Clinic Wisconsin having ~50% lower consent rates at 12 and 11%, respectively. A majority of consents were received within 30 days of initial invitation with 89% at MCR, 80% at MCF, and 81% at MCW. The rate of consent to MCB declined over time (Figure 1), with consent rates of 32–26% prior to 2011 decreasing down to 19–17% from 2013 to 2015.

**Figure 2:**
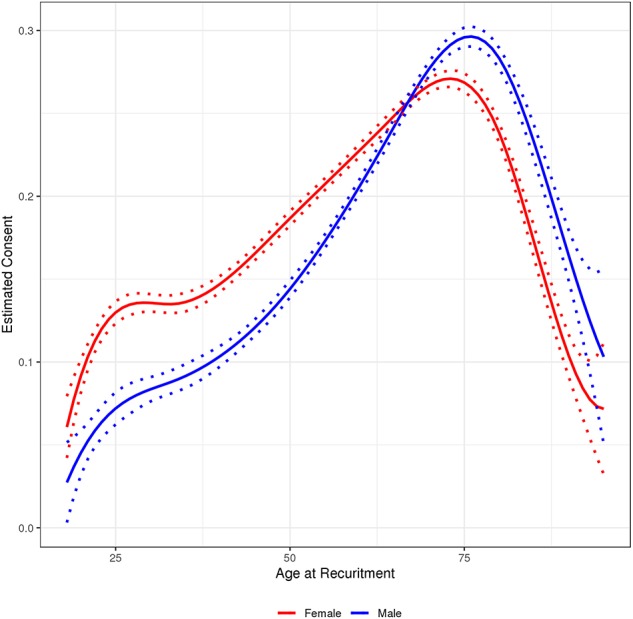
Age by gender interaction with 95% prediction interval for follow-up study consent rate among participants in the Mayo Clinic Biobank invited to participate in follow-up studies, 2010 through 2018.

The highest rate of consent was seen among the 70–89 year olds with 26% of patients consenting across the three sites. In MCR, the consent rate for 70–89 year olds was 32% and the consent rates for 50–69 year olds was 28%. MCF and MCW had substantially lower consent rates at these ages, 12% at both sites (Table A1). Within each of the three sites, consent rates were similar for males and females with an overall consent rate of 19% for both genders. However, we noted a significant interaction between age and gender. As shown in Figure 2, male and female consent rates steadily increased across age until the age of 78 years and then began to decline. Females had a significantly higher consent rate than males until the age of 65, at which time the male consent rate became higher (Figure 2). Patients who self-reported their race as white were most likely to consent at all sites (20% overall) with American Indian/Alaskan Native patients consenting at the second highest levels at 13% overall. Patients who self-reported as Asian or Black/African American consented at lower rates than any other group, at 10 and 7%, respectively. A slight difference in consent rates was noted between patients who lived 30–99 miles from their recruitment center and patients who lived 100 miles or more from their recruitment sites, 21 and 23%, respectively. We also evaluated whether time of year affected consent rates and saw that patient consent rates were lowest when patients were invited in November and December and highest in March, April, and October.

### Follow-Up Studies Participation

Of the 57,041 patients consented to the Biobank over the lifetime of the recruitment, 33,487 (59%) have been invited to participate in at least one follow-up study between 2009 and 2018 (Table 1). The median number of follow-up studies to which patients were invited was 2 (IQR: 1, 3) but ranged as high as 10. Among those invited to at least one follow-up study, 71% of subjects agreed to participate in at least one follow-up study and 30% participated in more than one study. Ninety-three percent (93%) of patients invited to follow-up studies were white, 60% were female, and 34% had some college education. These distributions were not meaningfully different from the overall Mayo Clinic Biobank population (Table 1). About 90% of follow-up study patients were recruited from patients enrolled at the Rochester site even though that group comprised only 77% of the MCB. This was due in large part to requests in many follow-up studies for geography-matched controls for their Rochester-based case-control studies.

**Table 1:** Demographic characteristics among Mayo Clinic Biobank participants invited to follow-up studies compared to the underlying MCB population from which they were selected.

	**Follow-up study**	**Biobank cohort**
	**(*N* = 33,487)**	**(*N* = 57,041)**
Female	19,952 (60%)	33,515 (59%)
White	30,741 (93%)	51,588 (92%)
Age at Biobank enrollment	57.1 (15)	59.5 (16)
Recruitment center		
Florida	2,387 (7%)	10,412 (18%)
Wisconsin	925 (3%)	2,659 (5%)
Rochester	30,175 (90%)	43,970 (77%)
Education		
High school or less	5,380 (16%)	9,137 (16%)
Some college	11,192 (34.0%)	18,464 (33%)
4 year degree	8,452 (26%)	13,998 (25%)
Advanced degree	7,965 (24%)	14,218 (26%)

The type of follow-up studies conducted influenced the participation rates (Table 2). Of the 43 follow-up studies considered, 33 included a request for identifier release from participants, 13 included a request for additional questionnaire data, and 16 included a request for additional biological samples (Table 2). Twenty-two studies included more than one type of request from the participants with 11 asking for both new samples and identifier release. Participation rates were highest in studies requesting additional questionnaires with a weighted mean participation rate of 69% (SD: 10%). Follow-up studies that requested identifier release or new samples had very similar participation rates, 56% (SD: 13%) and 54% (SD: 13%), respectively (Table 2). The limited number of studies that requested stool or urine samples had the lowest consent rates ranging from 14 to 45%. Seven of the follow-up studies above involved sequencing data, such as pharmacogenomics or genome wide association studies. The weighted mean consent rate to these studies was 56% (SD: 6%) and ranged from 14 to 85%.

**Table 2:** Overview of follow-up study participation rates by type of follow-up study data request.

**Study type**	***N* studies** **(*N* = 43)**	**Inter quartile** **range**	**Range** **(consent rate)**	**Mean consent** **rate (weighted)**
Identifier release	33	49–67%	13–85%	56% (SD: 13%)
New samples	16	30–59%	14–85%	54% (SD: 16%)
Blood	12	42–65%	19–85%	58% (SD: 13%)
Blood and urine spot	2	–	–	45%, 20%
24 h urine	1	–	–	14%
Stool	1	–	–	34%
Questionnaire	13	61–73%	57–87%	69% (SD: 10%)
Genetic studies	7	43–59%	14–85%	56% (SD: 6%)

*Many follow-up studies included more than one type of request and are included multiple times in the table above. Consent weights are weighted by sample size*.

Table 3 presents weighted mean consent rates across the various types of follow-up studies. We saw no meaningful difference in the consent rates of males (58%) and females (59%) to follow-up studies. Non-white patients were significantly less likely to participate in follow-up studies than white patients, 46 and 59%, respectively. For each age group, we saw an ~10% increase in the participation rates up to 49 years of age; a significant decrease was observed in participants who are over the age of 90, which is similar to the consent rates to the MCB. A similar age/gender interaction observed for MCB consent rates was also seen in the follow-up study participation rates. The follow-up study response rate was 53% among those who had been consented to MCB between 3 and 10 years earlier. In contrast, the response rate was 80% among those who had consented to MCB <12 months earlier. Participants with more education tended to participate more frequently in follow-up studies than those with less education (Table 3). These trends were consistent across follow-up study types.

**Table 3:** Weighted mean participation rates across various demographic factors by follow-up study type among Mayo Clinic Biobank participants invited to participate in follow-up studies, 2010 through 2018.

	**ID** **releases**	**Additional** **samples**	**Questionnaire** **requests**	**All follow-** **up studies**
Gender				
Female	56 (2.3)	55 (4.3)	69 (2.7)	59 (2.2)
Male	55 (2.5)	53 (3.8)	68 (3.0)	58 (2.3)
Race				
Non-white	43 (2.3)	39 (3.5)	57 (3.8)	46 (2.3)
White	57 (2.3)	55 (4.1)	70 (2.8)	59 (2.2)
Age at Invitation				
18–29	43 (3.3)	37 (5.0)	52 (4.9)	42 (2.9)
30–49	51 (2.0)	49 (3.8)	62 (3.3)	52 (2.0)
50–69	58 (2.7)	57 (4.4)	70 (2.8)	61 (2.4)
70–89	58 (2.8)	61 (5.2)	72 (3.2)	62 (2.7)
90+	25 (3.8)	37 (5.2)	49 (6.0)	37 (4.4)
Time from MCB consent				
<1 year	70 (2.7)	59 (3.5)	84 (1.7)	80 (1.9)
1–3 years	63 (2.2)	55 (4.5)	70 (2.4)	63 (1.9)
3.1–10+ years	52 (2.2)	53 (4.2)	62 (1.4)	53 (2.00)
Education				
High school or less	48 (2.3)	48 (3.3)	63 (3.6)	52 (2.3)
Some college	53 (2.2)	53 (4.2)	67 (2.8)	56 (2.2)
4 year degree	58 (2.4)	56 (4.4)	71 (2.7)	60 (2.3)
Advanced degree	62 (2.5)	59 (4.2)	74 (2.5)	65 (2.3)

*Sample size weighted means and standard deviations are shown*.

## Discussion

The goals of this current analysis were to investigate factors associated with consent rates to the Mayo Clinic Biobank as well as with willingness to participate in additional research projects through involvement in follow-up studies. We found that consent rates to the Mayo Clinic Biobank were associated with year of consent, declining from 32% in 2009 to 17% in 2015 with an overall consent rate of 19%. These rates are higher than what was observed for similar biobanks without a specific disease focus, such as the UK Biobank, which reported a 5.5% consent rate (5). The MCB's recruitment was targeted to patients with upcoming clinical appointments which is likely a large contributing factor to the improved consent rates compared to the UK Biobank. When comparing our consent rate to those seen in disease specific registries, MCB rates were much lower than the 32% reported across 37 disease-specific collections (6).

Disease registries have the added benefit of perceived patient engagement as patients presumably have more of a vested interest in advancing research for conditions for which they or family members suffer. The reason for the downward trend of consent rates over time is not fully understood. One possible explanation is that patients who were likely to participate were captured early in the recruitment process. However, when recruitment was expanded to MCF and MCW we observed participation rates similar to concurrent rates observed in MCR. There were no protocol or recruitment changes that explain the observed trend. This suggests that there may be larger environmental factors influencing consent rates over time. We found that males and females had comparable consent rates as a main effect; however, we noted an age by gender interaction. Other recruitments have shown various differences in consent rates for males and females with 7% increase in female consent shown for a sleep medicine trial (7) and the UK Biobank reporting a 5% consent rate for males and 6.5% for females (5). Observing differences in consent across males and females by age can be insightful in managing recruitment. The phenomena of young patients consenting at lower rates than older patients was expected, but the increased consent for older males was unexpected. This could be due to the increased health awareness in older males compared to young males. This trend suggests accounting for these expected differences during recruitment would allow studies to better balance their final cohorts.

One of the primary strengths of biobanks such as the MCB has been the ability to invite the participants to take part in additional follow-up studies. We have observed that MCB patients respond to these follow-up studies at rates higher than seen in patient populations external to the MCB. No publications could be found outlining rates of participation to additional follow-up studies in patients from non-disease-specific biobanks. We found that consent rates to follow-up studies followed the same downward trends over time noted in the rates of consent to the MCB. These trends seem to be fairly uniform across recruitment site. Of note is the time point between initial consent and invitation to additional follow-up studies. The greater the time since initial consent the less likely patients were to consent to follow-up studies. Participants were most likely to consent to additional follow-up studies within a year of initial MCB consent. The downward trend appears to stabilize from 3 to 10 years with a weighted mean consent rate of 53%. However, we will continue to monitor this trend over the lifetime of the MCB, as a continued downward trend may make recruitment less viable.

A limitation of this study is the relatively low frequency of non-white subjects enrolled to the MCB. This is being addressed by development of two separate biobanks focused on non-white populations, including the Sangre Por Salud Biobank (8) which targeted Latino and Latina patients, and Biobank Mississippi, which is enriched for persons with African descent (unpublished). Another limitation is the low response rates to the Mayo Clinic Biobank that would suggest that generalizations to other populations should be done cautiously.

The type of request in each follow-up study was important. Although we have had only a small number of studies requesting patients provide urine or stool samples, these follow-up studies had lower participation rates than other types of follow-up studies.

Several of the follow-up studies included some sort of genetic component. These studies included sequencing patient samples with potential return of results and we had expected lower rates of participation in these types of follow-up studies. However, we saw no meaningful differences in patient consent rates for these studies compared to other types of follow-up studies. One concern throughout was the differences in consent across the various racial groups. Not only did we see lower rates of consent to the overall Biobank in the non-white populations, but we saw lower rates of consent to follow-up studies in the same groups even among persons that had already consented to take part in the Mayo Clinic Biobank. This suggests that we may need to utilize different methods among these groups in future recruitment efforts, even for follow-up studies.

## Conclusion

In conclusion, we describe factors related to participation in the Mayo Clinic Biobank as well in studies embedded within it. We found that consent rates among younger and non-white patients were lower than in older, white patients. However, we also found that participation rates among those already enrolled in the biobank were much higher than those seen in new recruitment efforts, external to an existing biobank. We thus demonstrate an important way that biobanks can advance precision medicine goals: through provision of populations from which studies can draw participants for future studies.

## Data Availability Statement

To apply for access to data, contact Biobank@mayo.edu and request the current application for access to samples and/or data. Due to the specifics of the informed consent language, a Mayo Clinic researcher must be included as a collaborator on all projects.

## Ethics Statement

The studies involving human participants were reviewed and approved by Mayo Clinic Institutional Review Board. The patients/participants provided their written informed consent to participate in this study.

## Author Contributions

MH planned the manuscript with input from JO. MH and CK equally contributed to all drafts of the manuscript. JO, JC, ER, NL, JB, RG, LW, ST, and MC critically reviewed and updated the manuscript and approved the final version.

### Conflict of Interest

The authors declare that the research was conducted in the absence of any commercial or financial relationships that could be construed as a potential conflict of interest.

## Appendix

**Table A1:** Consent rates to MCB by recruitment center across basic demographic factors.

	**Consent rate (number invited)**			
	**FLA**	**LAC**	**RST**	**Total**
Total	12% (*N* = 69,628)	10.8% (*N* = 21,861)	24% (*N* = 159,613)	19.2% (*N* = 251,102)
Female	12% (*N* = 38,906)	10.8% (*N* = 14,475)	24% (*N* = 91,134)	19.2% (*N* = 144,515)
Male	12% (*N* = 30,722)	9.6% (*N* = 7,386)	22.8% (*N* = 68,479)	19.2% (*N* = 106,587)
Age at recruitment				
18–29	4.8% (*N* = 3,918)	4.8% (*N* = 2,616)	12% (*N* = 19,187)	9.6% (*N* = 25,721)
30–49	8.4% (*N* = 15,151)	7.2% (*N* = 6,004)	16.8% (*N* = 41,486)	13.2% (*N* = 62,641)
50–69	12% (*N* = 32,037)	12% (*N* = 9,815)	27.6% (*N* = 64,499)	21.6% (*N* = 106,351)
70–89	16.8% (*N* = 17,598)	15.6% (*N* = 3,281)	32.4% (*N* = 33,136)	26.4% (*N* = 54,015)
90+	8.4% (*N* = 924)	4.8% (*N* = 145)	12% (*N* = 1,305)	9.6% (*N* = 2,374)
Race				
American Indian/Alaskan Native	9.6% (*N* = 111)	12% (*N* = 24)	14.4% (*N* = 460)	13.2% (*N* = 595)
Asian	4.8% (*N* = 1,433)	3.6% (*N* = 188)	12% (*N* = 2,434)	9.6% (*N* = 4,055)
Black or African American	6% (*N* = 5,093)	3.6% (*N* = 226)	9.6% (*N* = 2,448)	7.2% (*N* = 7,767)
Choose Not To disclose	8.4% (*N* = 1,220)	0% (N = 3)	15.6% (*N* = 942)	12% (*N* = 2,165)
Native Hawaii/Pacific Islander	3.6% (*N* = 54)	9.6% (*N* = 10)	13.2% (*N* = 103)	9.6% (*N* = 167)
Other	6% (*N* = 669)	8.4% (*N* = 213)	13.2% (*N* = 2,369)	10.8% (*N* = 3,251)
Unknown	8.4% (*N* = 5,563)	7.2% (*N* = 705)	21.6% (*N* = 15,558)	18% (*N* = 21,826)
Unable to Provide	4.8% (*N* = 175)	4.8% (*N* = 61)	26.4% (*N* = 23)	7.2% (*N* = 259)
White	13.2% (*N* = 55,310)	10.8% (*N* = 20,431)	24% (*N* = 135,276)	20.4% (*N* = 211,017)
Distance from the clinic				
0–29 miles	10.8% (*N* = 37,300)	10.8% (*N* = 19,574)	21.6% (*N* = 72,974)	16.8% (*N* = 129,848)
30–99 miles	13.2% (*N* = 12,615)	10.8% (*N* = 2,063)	24% (*N* = 35,855)	20.4% (*N* = 50,533)
100–249 miles	13.2% (*N* = 11,552)	8.4% (*N* = 157)	27.6% (*N* = 19,730)	22.8% (*N* = 31,439)
250+ miles	13.2% (*N* = 8,160)	8.4% (*N* = 67)	25.2% (*N* = 31,239)	22.8% (*N* = 39,466)
